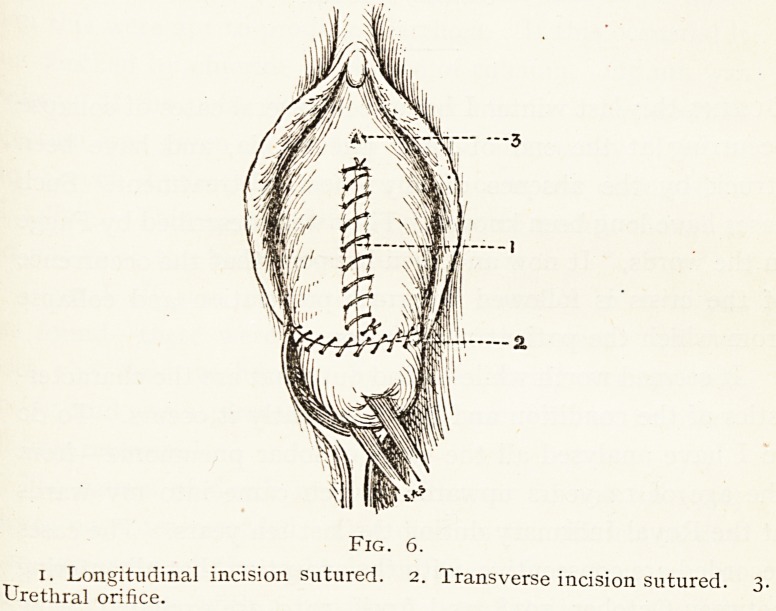# An Operation for the Cure of Prolapse and Cystocele

**Published:** 1920-06

**Authors:** Walter C. Swayne

**Affiliations:** Professor of Obstetrics in the University of Bristol; Obstetric Physician to the Bristol Royal Infirmary


*" Scire est nescire, nisi id me*
*Scire alius sciret."*

JUNE, 1920.

### AN OPERATION FOR THE CURE OF PROLAPSE AND CYSTOCELE.

BY

WALTER C. SWAYNE, M.D., B.S.,

*Professor of Obstetrics in the University of Bristol; Obstetric Physician to the Bristol Royal Infirmary.*

PROLAPSE and cystocele may occur either as separate entities, or both may be found in the same patient. The operative procedures described are applicable to either condition separately, to both when they occur together, or, in principle, but with certain modifications in detail, to rectocele.

The object of the procedure is to reconstruct the original anatomical relations of the parts concerned, or so to alter the anatomical relations existing as the result of the deformity or displacement that the physical effect of the original natural relations is reproduced. It is necessary before describing the operative procedures to consider for a moment the normal anatomy of the parts concerned and the alterations therein which lead to the production of the deformities mentioned.

It is unnecessary to describe in anatomical detail the construction of the upper part of the pelvic diaphragm beyond making the following statements :—

1. The levator ani with its fascial sheath (the sides of which are formed by the anal and pelvic fascial planes) is so attached and at such a level that in the erect posture it can have no possible function as a support of the uterus.

2. It does enter into the perineum, and at the point where its fibres divaricate to permit of the passage of the vagina, it can prevent the egress from the latter organ of any solid body which may be within it, *e.g.* a pessary.

3. The bases of the broad ligaments contain a comparatively large mass of connective tissue, the fibres of which follow the course of the uterine artery from the parietes to the uterus.

4. The floor of the bladder is separated from the anterior wall of the vagina by an interval which contains a felt-work of connective tissue (which, by the way, is exactly described by one of the meanings of the word *fascia*, a mat).

It should be stated with regard to 3 and 4 that the connective tissue elements described do *not* form either definite dissectible bands, such as the ordinary ligaments of the joints, or a definite dissectible plane, such as the *fascia lata* of the thigh, but a structure which may perhaps be likened to a soft woolly triangular bell-rope in the one case and a felted mat in the other.

The structures in 3 and 4 are more or less continuous, so that the connective tissue of this part of the pelvic floor would, if separated from the other soft parts (the uterus only being left *in situ*), resemble in outline the moon half obscured by a circular disc of more than her own diameter with an indentation in the centre of its intersecting arc.

The connective tissue (the two portions of which found between the layers of the broad ligaments at their bases accompanying the uterine vessels from their origin to the uterus and described as the ligaments of Patterson) *is* the actual support both of the uterus and the base of the bladder. The uterus is retained at its proper level in the pelvis by the so-called ligaments of Patterson, and the primary cause of prolapse of the uterus is, in the majority of cases, the stretching of these structures by parturition, while cystocele is due to a divarication, or one might say a hernial orifice in the felt-work of connective tissue between the bladder and the vaginal wall.

Basing the operative procedure on these facts, it has been my practice for the last seventeen years to deal with cases of prolapse and cystocele as follows :—

The cervix uteri is first grasped with vulsellum forceps and drawn outside the vaginal outlet.

A median incision through the vaginal mucous membrane is carried from a point about three-quarters of an inch behind the urethral orifice to the mid-point of the reflection of the vaginal mucous membrane from the cervix. The vaginal mucous membrane is then incised on each side of the cervix to a point just behind the mesial plane of the uterus, the incisions when completed forming a " T " with a curved top. (Fig. 1.)

Two lateral flaps are then dissected up roughly triangular in shape and attached by their longer sides.

The bladder is separated from the uterus as high as may be necessary to expose the bases of the broad ligaments, and the dissection carried onwards on each side until sufficient space is obtained. (Fig. 2.)

At this stage, if there is a well-marked cystocele, the bladder will be seen projecting through the hernial orifice in the felt-work connective tissue as a soft puckered mass. (Fig. 3.)

FIG. 1.

1. Volsellum on cervix. 2. Tenaculum grasping vaginal mucous membrane. 3. Longitudinal incision in vaginal wall. 4. Transverse incision round cervix.

FIG. 2.

1 and 2. Flaps raised and held by tenacula.

FIG. 3.

1, 1. Flaps as before. 2. Base of bladder protruding.

With a fully curved Hagedorn's needle, a thick catgut suture is passed through the base of the broad ligament on each side, usually from above downwards in each case. (Fig. 4.)

FIG. 4.

1, 1. Flaps as before. 2, 2. Needles carry suture through bases of broad ligaments. 3. Suture.

This suture is then firmly tied, with the result that the cervix is pushed backwards and upwards, and two folds of connective tissue can be seen running from just outside the tied suture to the back of the symphysis forming a well-marked triangle with its base at the cervix. These folds are then sewn together by means of a continuous suture of medium catgut, the bladder being pressed upwards with a flat retractor. (Fig. 5.) This suture can be reinforced if necessary by a second over it. The vaginal flaps are now brought together. After the ends have been cut off

FIG. 5.

1. Suture in bases of broad ligaments tied. 2. Running suture bringing folds of fibrous tissue together. 3. Base of bladder now pushed up. 4. Dotted lines marking edge of redundant flaps to be removed.

the deep sutures and any redundant portions cut away with scissors, the flaps are sewn together with either interrupted or continuous catgut sutures. (Fig. 6.)

The cervix may be amputated or the perineum repaired as the necessities of the case may demand; in the case of the former between the first and second steps of the procedure described, or on the completion of the procedure in the case of the latter.

This operation should not be performed on a woman likely to become pregnant, as parturition will almost certainly reproduce the original injury. Care must also be taken to avoid passing a suture round a ureter.

So far I have only had one failure to cure the original disability, and that was due to parturition at an unusually late age.

FIG. 6.

1. Longitudinal incision sutured. 2. Transverse incision sutured. 3. Urethral orifice.

Since I wrote the above an opportunity has occurred of examining after an abdominal section the conditions produced by this operation. It was found that there was a definite shelf right across the anterior half of the pelvis in front of the uterus. This shelf is produced by the tension put on the cellular connective tissue in the situations mentioned under (3) and (4).

The illustrations were sketched from life by Mr. A. Sewell.

## Figures and Tables

**Fig. 1. f1:**
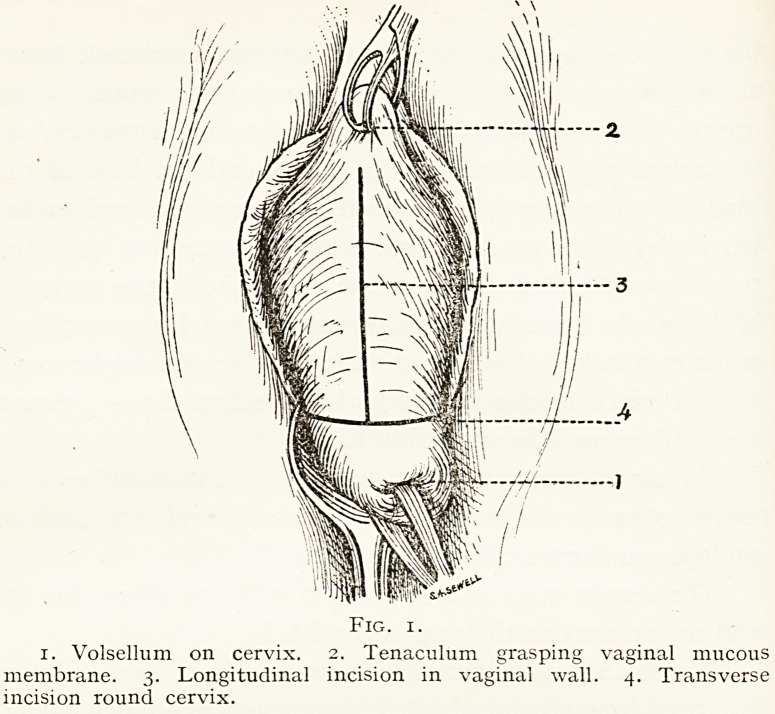


**Fig. 2. f2:**
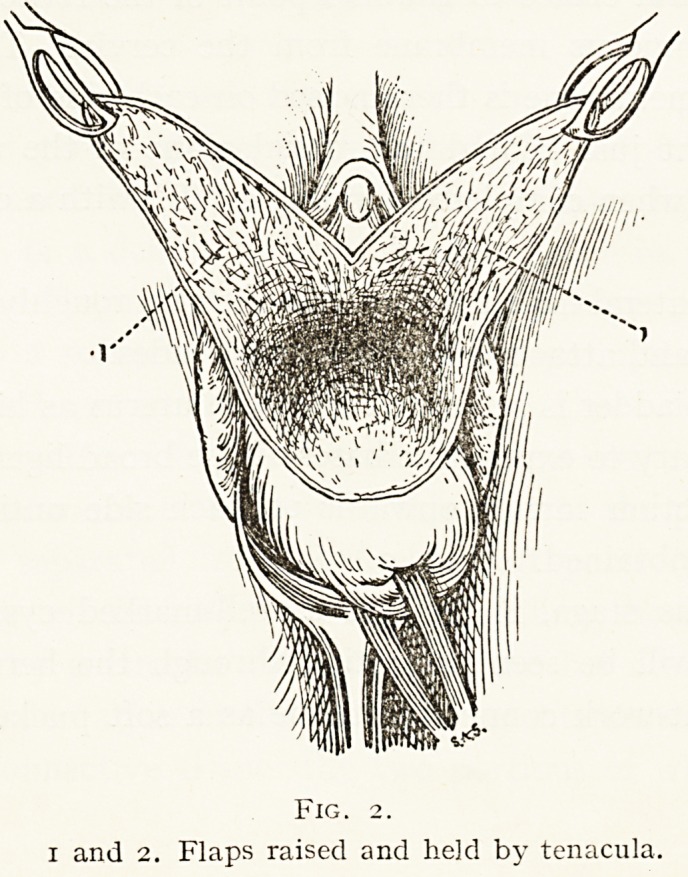


**Fig. 3. f3:**
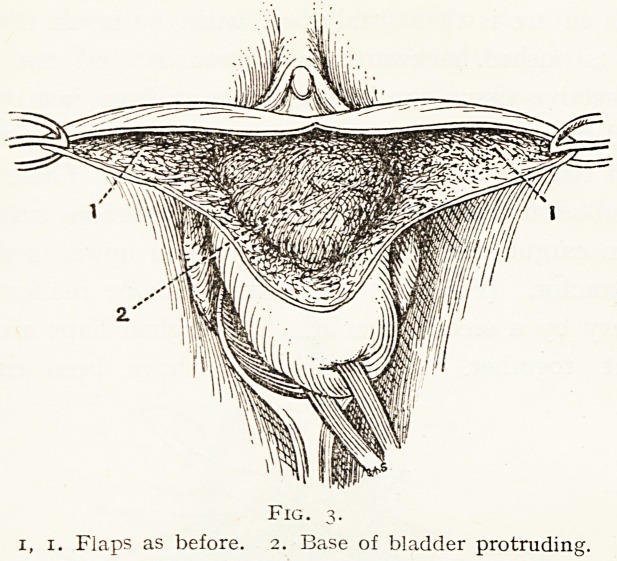


**Fig. 4. f4:**
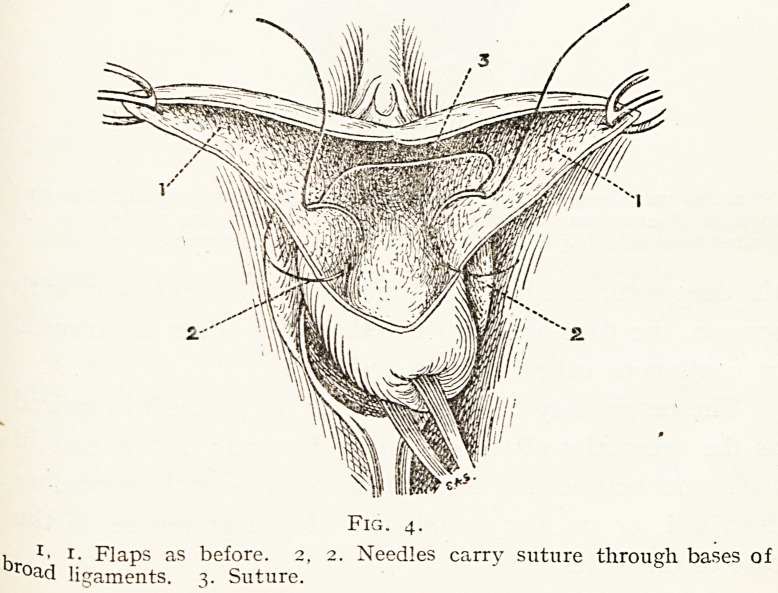


**Fig. 5. f5:**
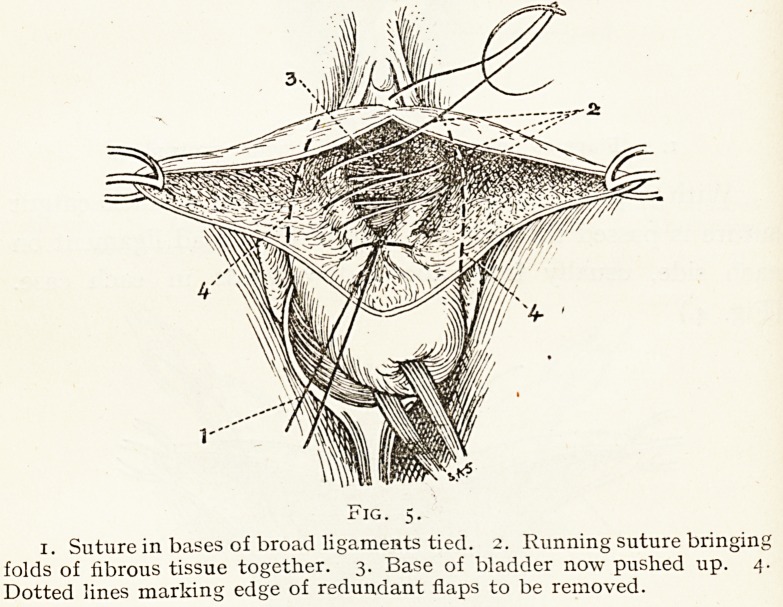


**Fig. 6. f6:**